# Genome-wide identification of calcineurin B-like protein-interacting protein kinase gene family reveals members participating in abiotic stress in the ornamental woody plant *Lagerstroemia indica*

**DOI:** 10.3389/fpls.2022.942217

**Published:** 2022-09-20

**Authors:** Chunmei Yu, Yongchao Ke, Jin Qin, Yunpeng Huang, Yanchun Zhao, Yu Liu, Hui Wei, Guoyuan Liu, Bolin Lian, Yanhong Chen, Fei Zhong, Jian Zhang

**Affiliations:** ^1^School of Life Sciences, Nantong University, Nantong, China; ^2^Key Laboratory of Landscape Plant Genetics and Breeding, Nantong University, Nantong, China

**Keywords:** CIPKs, *Lagerstroemia indica*, gene family, abiotic stress, overexpression, salt tolerance, Arabidopsis

## Abstract

Calcineurin B-like protein-interacting protein kinases (CIPKs) play important roles in plant responses to stress. However, their function in the ornamental woody plant *Lagerstroemia indica* is remains unclear. In this study, the *LiCIPK* gene family was analyzed at the whole genome level. A total of 37 *LiCIPKs*, distributed across 17 chromosomes, were identified. Conserved motif analysis indicated that all LiCIPKs possess a protein kinase motif (S_TKc) and C-terminal regulatory motif (NAF), while seven LiCIPKs lack a protein phosphatase interaction (PPI) motif. 3D structure analysis further revealed that the N-terminal and C-terminal 3D-structure of 27 members are situated near to each other, while 4 members have a looser structure, and 6 members lack intact structures. The intra- and interspecies collinearity analysis, synonymous substitution rate (*K*_*s*_) peaks of duplicated *LiCIPKs*, revealed that ∼80% of *LiCIPKs* were retained by the two whole genome duplication (WGD) events that occurred approximately 56.12–61.16 million year ago (MYA) and 16.24–26.34 MYA ago. The promoter of each *LiCIPK* contains a number of auxin, abscisic acid, gibberellic acid, salicylic acid, and drought, anaerobic, defense, stress, and wound responsive *cis*-elements. Of the 21 members that were successfully amplified by qPCR, 18 *LiCIPKs* exhibited different expression patterns under NaCl, mannitol, PEG8000, and ABA treatments. Given that *LiCIPK30*, the *AtSOS2* ortholog, responded to all four types of stress it was selected for functional verification. *LiCIPK30* complements the *atsos2* phenotype *in vivo*. 35S:LiCIPK-overexpressing lines exhibit increased leaf area increment, chlorophyll a and b content, reactive oxygen species scavenging enzyme activity, and expression of *ABF3* and *RD22*, while the degree of membrane lipid oxidation decreases under NaCl treatment compared to WT. The evolutionary history, and potential mechanism by which *LiCIPK30* may regulate plant tolerance to salt stress were also discussed. In summary, we identified *LiCIPK* members involved in abiotic stress and found that *LiCIPK30* transgenic Arabidopsis exhibits more salt and osmotic stress tolerance than WT. This research provides a theoretical foundation for further investigation into the function of *LiCIPKs*, and for mining gene resources to facilitate the cultivation and breeding of new *L. indica* varieties in coastal saline-alkali soil.

## Introduction

Stability of coastal ecosystems is vital for minimizing the destruction of sea winds and tides. However, high salt concentrations of coastal soil, as well as a deficiency or imbalance in inorganic nutrients, such as nitrogen (N), phosphorus (P), and potassium (K), can impact plant growth. Hence, plants have evolved various mechanisms to adapt to changes in their environment. Indeed, plants have evolved calcium signaling to regulate development, plant–microbe interactions, and environmental signal (e.g., abiotic stress) perception (Ghosh et al., 2021). More specifically, calcineurin B-like proteins (CBLs) and CBL-interacting protein kinases (CIPKs, a Ser/Thr protein kinase), are plant-specific calcium sensors with numerous functions. For instance, the salt over sensitive (SOS) pathway, which has four main components (SOS1, SOS2, SOS3, and SOS3-like calcium binding protein 8/SCaBP8), is a basic calcium signaling pathway that has been elucidated in higher plants under salt stress (Halfter et al., 2000; Liu et al., 2000; Guo et al., 2001; Gong et al., 2002; Qiu et al., 2002; Quan et al., 2007). In this pathway, AtSOS3/CBL10 (or SCaBP8) functions as a Ca^2+^ sensor, binding to AtCIPK24/SOS2 to form an active complex, it then phosphorylates SOS1—an Na^+^/H^+^ exchanger located on the cell membrane—to regulate Na^+^ exclusion by the cell. Additionally, other CIPKs, such as maize ZmCBL1/4-ZmCIPK42, regulate salt stress tolerance at the seedling stage (Chen et al., 2021). For example, GmPKS4, a soybean CIPK, regulates soybean responses to salt and alkali stresses (Ketehouli et al., 2021). Besides salt stress, a CaCIPK3 drought response cassette was identified in pepper (*Capsicum annuum* L.) (Ma et al., 2021). AtCIPK23, through combining different CBLs, regulates the uptake and homeostasis of different ions, such as nitrate nitrogen (${\mathrm{NO}}_{3}^{-})$, ammonium (${\mathrm{NH}}_{4}^{+}$), K^+^, Mg^2+^, and Fe^2+^ (Cheong et al., 2007; Ragel et al., 2015; Tian et al., 2016; Straub et al., 2017; Morales de Los Ríos et al., 2021; Ródenas and Vert, 2021). OsCIPK9 is a multifaceted kinase that responds to salinity, osmotic stress, and K^+^ deficiency in rice (Ketehouli et al., 2021). The CIPKs are also involved in root architecture formation for various plants, such as ZmCIPK15 in maize, *zmcipk15-*knockout mutant exhibited a steeper root growth angle, higher nitrogen absorption, and greater shoot biomass compared to the WT. Meanwhile, overexpression of chrysanthemum *CmCIPK23* in Arabidopsis significantly decreases lateral root number and length, primary root length, and nitrogen uptake (Liu et al., 2022). The *zmcipk42* mutant also has fewer branched tassels and reduced salt stress tolerance at the seedling stage (Chen et al., 2021). Additionally, *OsCIPK31* participates in the development of panicle apical spikelets (Peng et al., 2018). Generally, orthologous genes have similar functions among different species, such as the SOS2 orthologs among different plant species. However, certain examples have been reported, for example, the overexpression of *VaCIPK02* (Amur grape), the *AtCIPK6* ortholog, enhances salt sensitivity in Arabidopsis (Xu et al., 2020) while overexpression of chickpea (*Cicer arietinum*) *CaCIPK6* enhances salt tolerance (Tripathi et al., 2009). The sequence difference between orthologous proteins may lead to the recruitment of different partners or downstream targets. Therefore, functions of orthologs among different plants require further experimental verification.

Due to three common whole genome duplication (WGD) events of dicotyledonous plants (ζ, zeta seed plant-wide WGD; ε, epsilon angiosperm-wide WGD event; and γ, gamma triplicated of dicotyledon-wide WGD) (Tang et al., 2008; Clark and Donoghue, 2018; Li and Barker, 2020), the retention of CIPKs during the evolution of a plant and their role in plant adaption represents an interesting research direction. In fact, genome-wide analysis of the *CIPK* gene family in several plants, including Arabidopsis, rice, grape, *Prunus mume*, tea (*Camellia sinensis* var. Sinensis), and turnip (*Brassica rapa* var. *rapa*), has identified members participating in abiotic stress (Kolukisaoglu et al., 2004; Yu et al., 2007; Xi et al., 2017; Li et al., 2019). For example, in a horticultural/ornamental plant, *P. mume*, a total of 16 CIPK genes were identified. Twelve *PmCIPK* genes are up-regulated during cold stress treatment, implying that *PmCIPKs* may be involved in distribution of different *P. mume* varieties (Li et al., 2019). In turnip, 51 *BrrCIPK* genes have diverse expression patterns during development and different stimulation with several CIPKs found to have more than one CLB partner (Yin et al., 2017). These studies indicate that genome-wide analysis can identify stress-related *CIPKs* efficiently.

*Lagerstroemia indica* (crape myrtle) is an important ornamental shrub (tree) characterized by its long flowering period, different flower color, and bark trunks. It is also a traditional medicinal plant with several effective secondary metabolites (Yang et al., 2011; Labib et al., 2013). During the past decade, research has primarily focused on genes determining ornamental traits, such as leaf and flower color (Wang et al., 2017; Qiao et al., 2019; Li et al., 2020a; Yu et al., 2021), as well as the dwarf and weeping architecture (Ye et al., 2015; Li et al., 2020b). However, during its long life, *L. indica* faces several challenges, including the threat of diseases and soil stress (nutrient and water shortages, high pH, or high salt in some coastal areas). Studies have been undertaken to determine the pathogenicity of leaf spot and powdery mildew (Shi and Mmbaga, 2006; Babu et al., 2014; Kim, 2021), to identify genes resistant to powdery mildew (Wang et al., 2015). However, there are currently few reports on the response of *L. indica* to salt stress, the existence of salt-tolerant varieties, and genetic resources that could be used to improve salt stress tolerance during the breeding program. To our knowledge, the functions of *LiCIPKs* have not been reported yet; thus, rendering an incomplete understanding of the mechanism by which crape myrtle responds to external stress signals.

As CIPKs are the homeostat of several ions in plants, we sought to determine the roles of LiCIPKs in crape myrtle adaptation to abiotic stress. More specifically, we assessed *LiCIPK* gene family characteristics, evolutionary history, and expression patterns under several abiotic stress conditions, and defined their associated functions. Our objectives are to: (1) better understand the *L. indica* genome; (2) identify LiCIPK members that participate in abiotic stress; and (3) verify LiCIPK function. Our results provide a theoretical foundation for further functional analysis of CIPKs in plant adaptation to stress.

## Materials and methods

### Plant material, growth conditions and chemical reagents

Plant materials in this study include the *L. indica* var. Black Diamond ‘Blush V2’ and four genotypes of Arabidopsis, Col-0 (WT), *atsos2* mutant (T-DNA SALK_056101C), LiCIPK30/*atsos2* (COM) and LiCIPK30/WT (OE). All Arabidopsis lines were germinated on half-strength Murashige & Skoog media supplied with 2% sucrose (1/2 MS) for 10–14 days, then the seedlings were planted on mixed soil (50% Pindstrup Substrate and 50% vermiculite) and grown in an artificial climate chamber (16 h day/8 h night, 22°C day/18°C night).

The chemical reagents used in this study were purchased from SINOPHARM (Beijing, China) or Ameresco (Framingham, MA, United States). The RNA extraction reagent (MiniBEST Plant RNA Extraction Kit), first strand cDNA synthesis kit (PrimeScript™ RT reagent Kit with gDNA Eraser), and TB GREEN reagents for quantitative polymerase chain reaction (qPCR) were purchased from TaKaRa (Beijing, China). The plant genomic DNA extraction kit was purchased from TIANGEN (DP305, Beijing, China), and plasmid DNA extraction kit from Beyotime (Shanghai, China). Primers were synthesized by General Bio (China, Anhui, Chuzhou). All oligo primers used in this study are listed in Supplementary Table 1.

### Identification of *L. indica* calcineurin B-like protein-interacting protein kinase genes

The HMM files of the protein kinase (PF00069) and NAF motif (PF03822) were downloaded from the pfam protein database^1^. The candidate LiCIPKs in the *L. indica* genome (Accession number CNP0003018, unpublished data from our lab) were obtained by HMM search (*E*-value < 1e-5, Identity ≥ 50%) using the TBtools software (V1.098689) (Chen et al., 2020). Candidates were also aligned using BLAST against the AtCIPKs (Kolukisaoglu et al., 2004) and filtered according to the methods described by Zhang et al. (2021). The distribution of *LiCIPKs* on chromosomes was depicted by three files (chromosome length, Gene ID, and GFF3) using TBtools (Chen et al., 2020). The theoretical isoelectric point (pI) and molecular weight (MW) of LiCIPKs were predicted using ExPASy^2^. Subcellular location was estimated using Wolf PSORT^3^ and SignalP^4^.

### Gene structure and conserved motifs analysis

The intron--exon structures of *LiCDPK*s were obtained using the genome sequences of *L. indica*, GFF3 files, and CDS sequences of all *LiCDPKs*. The conserved LiCDPK sequences were analyzed using MEME program with the parameters: motif 10 and width between 6--100 amino acid residues^5^. Graphic of genes structure and conserved motifs were drawn using TBtools software (Chen et al., 2020).

### Three-dimensional (3D) structure prediction of LiCIPKs

The 3D structures of LiCIPKs were predicted on the SWISS-MODEL website through homologous modeling^6^. The pdb files were opened using chimera soft^7^ and the structure of LiCIPKs was compared to that of AtCIPK24/SOS2 reported previously (Sánchez-Barrena et al., 2007; Chaves-Sanjuan et al., 2014).

### Calcineurin B-like protein-interacting protein kinase phylogenic tree construction

Arabidopsis AtCIPKs (*Arabidopsis thaliana, At*), rice OsCIPKs (*Oryza sativa, Os*), and grape VvCIPKs (*Vitis vinifera, Vv*) were extracted from their genomes according to previous reports (Kolukisaoglu et al., 2004; Yu et al., 2007; Kanwar et al., 2014; Xi et al., 2017). The amino acid sequences of CIPKs from four plants were used to construct a neighbor-joining (NJ) phylogenetic tree by MEGA X soft using default parameters (Kumar et al., 2017). The phylogenic tree was designed by web-based soft Evolgenius^8^ (Zhang et al., 2012; He et al., 2016; Subramanian et al., 2019).

### The synteny of calcineurin B-like protein-interacting protein kinase loci among three species

Synteny of CIPK gene loci between Arabidopsis, *L. indica*, and grape (*V. vinifera*) was analyzed using the One Step MCScanX in TBtools. Cognate loci intra *L. indica* was analyzed using the advanced Circos in TBtools.

### Divergence time calculation of duplicated genes

After obtaining duplicated gene pairs, the synonymous substitution rate (*K*_*s*_) and non-synonymous substitution rate (*K*_*a*_) of gene pairs were calculated using the “Simple *K*_*a*_/*K*_*s*_ calculator” tool of TBtools. Based on the *Lythraceae* specific rate (λ) of 1.14 × 10^–8^ substitutions per site per year (Feng et al., 2021), the divergence time (million years ago, MYA) of duplicated gene pairs was calculated according to the formula *T* = *K*_*s*_/2λ. Two common rates, 1.5 × 10^–8^ or 6.1 × 10^–9^, were also used as references (Lynch and Conery, 2000; Blanc and Wolfe, 2004).

### *Cis*-elements analysis of *LiCIPK* promoters

*Cis* elements in the promoter (−2,000 bp upstream ATG) were predicted on the PlantCARE website^9^. A combined diagram of LiCIPK phylogenic tree and *cis*-element distribution was drawn using the TBtools software (Chen et al., 2020).

### Stress treatment of *L. indica*

*L. indica* var. Black Diamond ‘Blush V2’ was used in this study. Under normal conditions, the semi-hardwood healthy branches were cut into 10–12 cm long pieces with at least four axillary buds to form cuttings, which were sterilized using 0.0625% carbendazim for 15–20 min, and subsequently planted in vermiculite soil in pots. All cuttings were grown in a greenhouse at 25 ∼ 35°C, under 16 h day/8 h night conditions (2020–2021). When new adventitious roots had grown to a length of ∼2–3 cm (at least 45 days after planting), they were treated with irrigation water containing 200 mmol L^–1^ NaCl, 15% PEG8000 and 10 × 10^–4^ mol L^–1^ abscisic acid (ABA), or 200 mmol L^–1^ mannitol to induce salt, drought, or osmotic stress (three biological repeats each), respectively (Liu X. et al., 2020). After treatment for 0, 1, 2, 3, 4, 5, and 6 days, adventitious roots were collected for RNA extraction, performed on the same day.

### RNA extraction and cDNA synthesis

RNA from the roots of all stress-treated materials was extracted using the MiniBEST Plant RNA Extraction Kit (TaKara). The RNA was converted to first strand cDNA using the PrimeScript™ RT reagent Kit with the gDNA Eraser kit. All procedures were performed according to the manufacturer’s instructions.

### Real-time quantitative PCR

Primers used for qRT-PCR are listed in Supplementary Table 1. The program was performed using ABI7500 according to the manufacturer’s instructions. PCR mixes were made following the protocols of the TB GREEN kit. The expression levels were calculated using 2^–ΔΔ*Ct*^ compared to the internal control and CK sample (Yu et al., 2021).

### Cloning of *LiCIPK30* and vector construction

Leaf RNA of *L. indica* and first-strand cDNA were obtained using the methods described above. *LiCIPK30* was amplified by 2 × Pfu MasterMix (CWBIO, CW0686, Beijing, China) using the primers listed in Supplementary Table 1. The PCR products were cloned into a pGEMT-T easy vector (Promega, Shanghai, China), according to the manufacturer’s protocols. The pWM101-*35S:LiCIPK30* was constructed using an in-fusion strategy (ClonExpress II One Step Cloning Kit, C112-01, Vazyme, Nanjing, China).

### Transformation of Arabidopsis

The pWM101-*35S:LiCIPK30* construct was first transformed into Arabidopsis WT (Col-0) and at*sos2* mutants through agrobacterium-mediated (GV3101) floral dip method reported previously (Clough and Bent, 1998). The positive LiCIPK30/*atsos2* (COM) and LiCIPK30/WT (OE) T1 plants were screened using half strength (1/2) MS with 20 mg L^–1^ hygromycin and genomic PCR with *LiCIPK30-*specific primers (Supplementary Table 1).

### Salt and mannitol stress treatment of Arabidopsis

The WT, *atsos2*, T3 Arabidopsis lines of COM and OE were treated at two different developmental stages. At germination, the seeds were planted in 1/2 MS medium with 0, 75, and 125 mmol L^–1^ NaCl or 0, 100, and 200 mmol L^–1^ mannitol. Root length of different lines was observed 10 days after planting. At the rosette stage (6–8 full-size leaves), the plants were treated by irrigating with 0, 100, and 200 mmol L^–1^ NaCl solution. The leaves were harvested for further analysis after 0, 3, and 7 days of treatment. All treatments were applied to at least three replicates.

### Detection physiological parameters

Leaves of WT and two independent LiCIPK30 lines were homogenized using liquid nitrogen. The activity of catalase (CAT, A007-1-1), and peroxidase (POD, A084-3-1), as well as the content of malondialdehyde (MAD, A003-3-1), and chlorophyll a and b (A147-1-1) were detected using the respective kits according to the manufacturer’s instructions (NJJCBIO, Nanjing, China). All treatments were applied to at least three replicates.

### Data analysis

Graphs of the data were constructed using Origin 2018 (Originlab, MA, United States). Differences between treatments or genotypes were analyzed on SPSS23.0 (IBM SPSS, NY, United States) using *t*-test or two-way analysis of variance (ANOVA).

## Results

### Identification and characterization of LiCIPKs

LiCIPKs were identified through HMM and BLAST search using the amino acid sequence of 26 AtCIPKs. After filtering the amino acid sequences based on length limits, a total of 37 full length proteins with a NAF domain and high sequence similarity to AtCIPKs were identified as LiCIPK family members. The name, chromosome location, peptide length, MW, and subcellular locations are listed in Table 1. The theoretical pI varied from 6.11 (LiCIPK3) to 9.33 (LiCIPK21), indicating different residues on the protein surface which may recruit different partners *in vivo*. LiCIPKs were predicted to localize to the cytoplasm (7 members), nuclear compartment (5 members), chloroplast (20 members), endoplasmic reticulum (1 members), and cytoskeleton (1 member). This variable distribution of LiCIPK family members implies that they may be involved in multiple biological processes (Table 1).

**TABLE 1:** Characteristic of LiCIPKs.

Subgroup	Gene name	Gene ID	Chr	AA length	PI	MW (KDa)	Predicted localization
Group Aa	*LiCIPK18*	evm.model.Chr8.403.2	8	452	8.03	51.59	Chl
	*LiCIPK23*	evm.model.Chr11.440	11	439	6.46	50.38	Chl
	*LiCIPK31*	evm.model.Chr15.863	15	439	6.86	50.43	Chl
	*LiCIPK35*	evm.model.Chr22.156.2	22	464	8.99	53.11	Chl
	*LiCIPK36*	evm.model.Chr23.882	23	439	6.56	50.17	Chl
Group Ab	*LiCIPK9*	evm.model.Chr4.1714	4	449	8.74	50.42	Chl
	*LiCIPK25*	evm.model.Chr12.477	12	465	8.94	51.5	Chl
Group Ac	*LiCIPK26*	evm.model.Chr12.934	12	448	6.69	50.86	Cyt
	*LiCIPK16*	evm.model.Chr7.12	7	446	9.07	50.31	Mit
	*LiCIPK30*	evm.model.Chr15.86.2	15	492	8.94	55.46	Chl
Group Ad	*LiCIPK8*	evm.model.Chr4.815	4	454	7.55	50.85	Chl
	*LiCIPK11*	evm.model.Chr5.959	5	443	6.22	50.09	Cyt
Group Ae	*LiCIPK2*	evm.model.Chr2.353	2	436	6.87	49.04	Nuc
	*LiCIPK3*	evm.model.Chr3.750.1	3	467	6.11	52.81	Chl
	*LiCIPK17*	evm.model.Chr7.317	7	436	6.65	48.9	NuC
	*LiCIPK29*	evm.model.Chr15.77	15	466	7.06	51.56	Nuc
Group B	*LiCIPK1*	evm.model.Chr2.66	2	436	8.93	47.8	Chl
	*LiCIPK5*	evm.model.Chr3.1533	3	432	9.1	47.58	Chl
	*LiCIPK27*	evm.model.Chr13.774	13	435	9	48.14	Chl
	*LiCIPK34*	evm.model.Chr21.370	21	433	9.2	47.84	Chl
Group Ca	*LiCIPK6*	evm.model.Chr4.21	4	448	9.13	50.53	Chl
	*LiCIPK14*	evm.model.Chr5.1704	5	448	8.86	50.42	Cyt
Group Cb	*LiCIPK4*	evm.model.Chr3.994	3	442	9.04	49.53	Cyt
	*LiCIPK24*	evm.model.Chr11.1405	11	443	8.8	49.74	PM
	*LiCIPK28*	evm.model.Chr15.32	15	450	9.06	50.54	Chl
Group Cc	*LiCIPK15*	evm.model.Chr6.1537	6	461	8.8	52.26	Cyt
	*LiCIPK33*	evm.model.Chr17.458	17	448	9.04	50.96	Cyt
Group Cd	*LiCIPK37*	evm.model.Chr24.188	24	462	9.02	52.01	Chl
	*LiCIPK21*	evm.model.Chr9.1257	9	335	9.33	38.29	Chl
	*LiCIPK7*	evm.model.Chr4.131	4	466	9.2	52.61	Nuc
	*LiCIPK13*	evm.model.Chr5.1568	5	462	9.01	52.1	Chl
Group D	*LiCIPK12*	evm.model.Chr5.1567	5	441	8.39	49.46	Cyt
	*LiCIPK20*	evm.model.Chr9.1255	9	451	9.13	50.9	Cyt
	*LiCIPK22*	evm.model.Chr11.4	11	440	9.09	49.56	Nuc
	*LiCIPK32*	evm.model.Chr15.1211	15	439	8.89	49.31	CytS
Group E	*LiCIPK10*	evm.model.Chr5.724	5	476	8.6	53.47	ER
	*LiCIPK19*	evm.model.Chr9.246	9	476	8.46	53.62	Chl

Chl, chloroplast; ER, endoplasmic reticulum; Nuc, nucleus; Mit, mitochondria; Cyt, cytoplasm; CytS, cytoskeleton; PM, plasma membrane. MW, molecular weight. pI, isoelectric point. Groups of the *LiCIPKs* were divided by results of phylogenic analysis (Figure 3).

**FIGURE 1:**
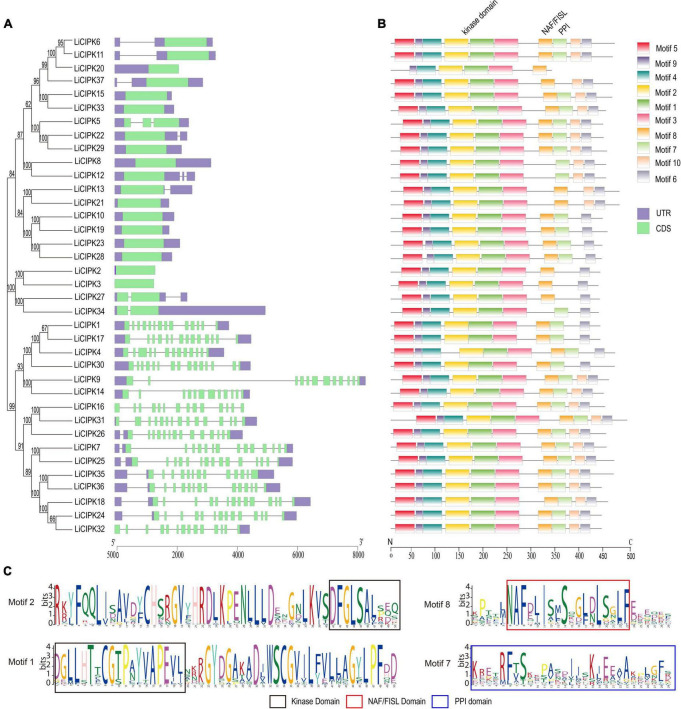
Phylogenetic tree, conserved motifs, and gene structure of *LiCIPKs*. **(A)** Exon–intron patterns. **(B)** Conserved motifs arrangement of LiCIPKs. **(C)** The functional domains. Motifs and exons are indicated as rectangles, other sequences as lines.

From the exon–intron patterns of *LiCIPKs*, we found 21 *LiCIPKs* to be intron-less (two introns or less) accounting for 56.76% of the total CIPKs. All others were intron-rich (containing 11–14 introns) genes. The exon-intron patterns were similar to that of the *VvCIPKs* of grape and *CsCIPK* of tea (Yin et al., 2017; Liu et al., 2019) (Figure 1A), implying that the gene structure of CIPKs diverged before the evolution of these species.

**FIGURE 2:**
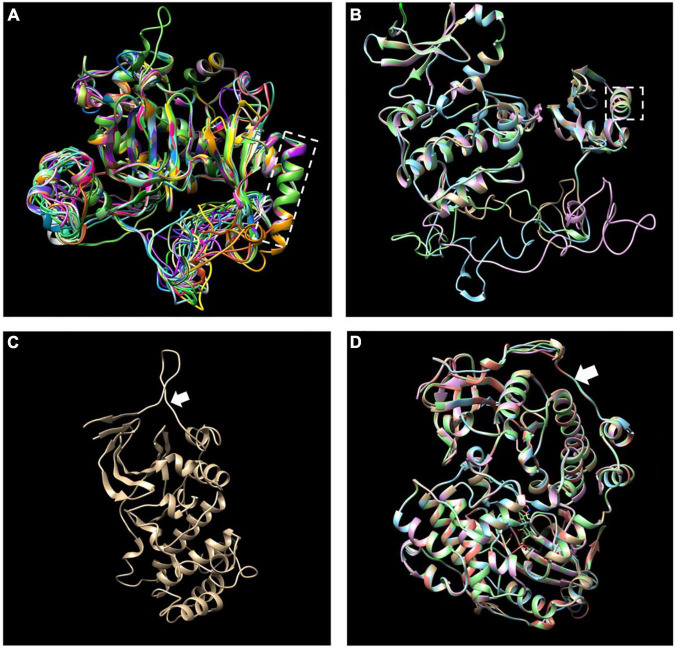
Merged three-dimensional (3D) structure of 37 LiCIPKs. **(A)** Merged 3D structure of LiCIPKs belong to class A to E. **(B)** Merged structure of LiCIPKs belong class F. **(C)** 3D structure of class G. **(D)** Merged structure of LiCIPKs class H. The classes A to H are depicted on Supplementary Figure 2. The dashed box indicates the α-helix of PPI domain; arrow indicates the junction between the N and C-terminal.

The MEME motif analysis showed that most LiCIPks had ten conserved motifs, including motifs 1 and 2 in the N-terminal kinase domain, which are in all LiCIPKs (Figure 1B and Supplementary Figure 1), and the C-terminal-regulating NAF/FISL domain in motif 8, which was identified in 35 LiCIPKs. This result differs from that of the multiple-alignment of the N-end and C-end active domains, which indicates that all CIPKs possess NAF/FISL sequences (Figures 1B,C and Supplementary Figure 1). Motif 7 is the protein phosphatase interaction (PPI) domain, which exists in 30 LiCIPKs (Figure 1 and Supplementary Figure 1). To elucidate whether less conserved sequences of motifs 7 and 8 affect the 3D structure, we used a homologous-based model from the expasy website (see Text Footnote 6). The results showed that the 37 LiCIPKs could be divided into eight classes according to their three-dimensional structure (Figure 2 and Supplementary Figure 2). Classes A to E are compact, with the N- and C-ends adjacent to each other (Figure 2A and Supplementary Figures 2A–E). The major differences between classes A to E are the α-helix numbers and arrangements ahead of NAF/FISL domain compared to that of LiCIPK24/SOS2 (Figure 2A and Supplementary Figures 2A–E). The 3D structure of class F is looser, with the N-end far away from the C-end (Supplementary Figure 2F and Figure 2B), similar to the active structure model of AtCIPK24 (Chaves-Sanjuan et al., 2014). The 3D structure of classes G and H only contain the N-terminal sequence, however, the 3D structure of class H is the homodimer of N-terminal sequences (Figures 2C,D and Supplementary Figures 2G,H). Of the CIPKs lacking motif 7 (e.g., LiCIPK1, -5, -19, -27, and -37), although the sequences are less conserved (Figure 1), they fold into a PPI α-helix (Supplementary Figures 2A,C,E,F). Only LiCIPK10 lacked the C-terminal structure (except LiCIPK21 with C-terminal sequence deletion; Figure 1). In summary, we found that the 3D structure analysis provided additional details on the active domain compared to the conserved sequences analysis.

### Phylogenic analysis of LiCIPKs

**FIGURE 3:**
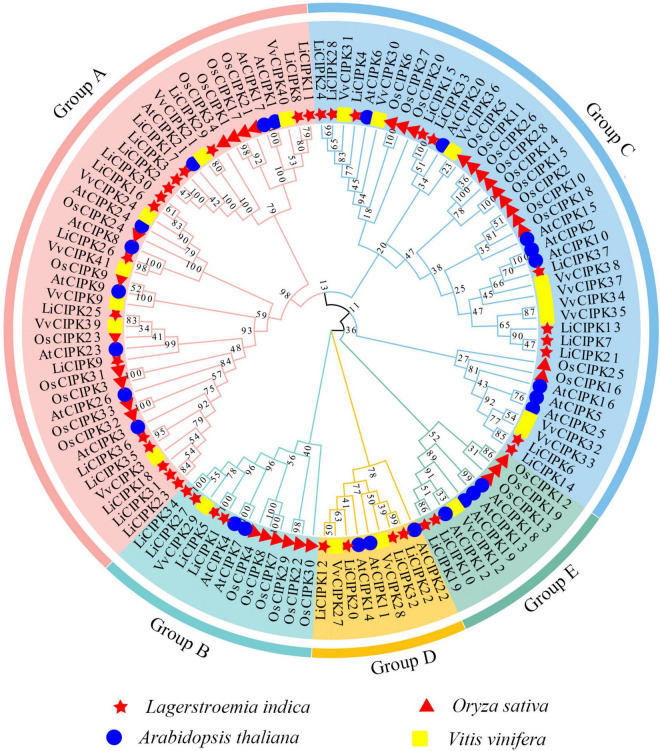
NJ-Phylogenic tree of AtCIPKs, OsCIPKs, VvCIPKs, and LiCIPKs. The group A to E is shaded by Jade red, Mint cyan, wathet blue, orange, and aquatic green, respectively. Genes of *L. indica* (*Li*), *Arabidopsis thaliana* (*At*), *Oryza sativ*a (*Os*), and *Vitis vinifera* (*Vv*) are marked by red star, blue circle, red triangle, and yellow rectangle, respectively.

To reveal the phylogenic relationship of the LiCIPKs, an NJ phylogenetic tree was constructed using the full-length amino acid sequences of CIPKs from *L. indica* and three other species (Arabidopsis, rice, and grape). A total of 117 CIPKs were divided into five groups (A–E) (Figure 3 and Supplementary Table 2). Of these, group A was the largest, containing 44 members and group D was the smallest with only 9 members. We found that groups A and C could be further divided into several subgroups (Figure 2 and Supplementary Table 1). Generally, the evolutionary relationship between *LiCIPKs* and *VvCIPKs* is closer than that between *LiCIPKs* and *AtCIPK* or *OsCIPKs*. Hence, the evolutionary rate of the LiCIPK gene family is faster than predicted.

**FIGURE 4:**
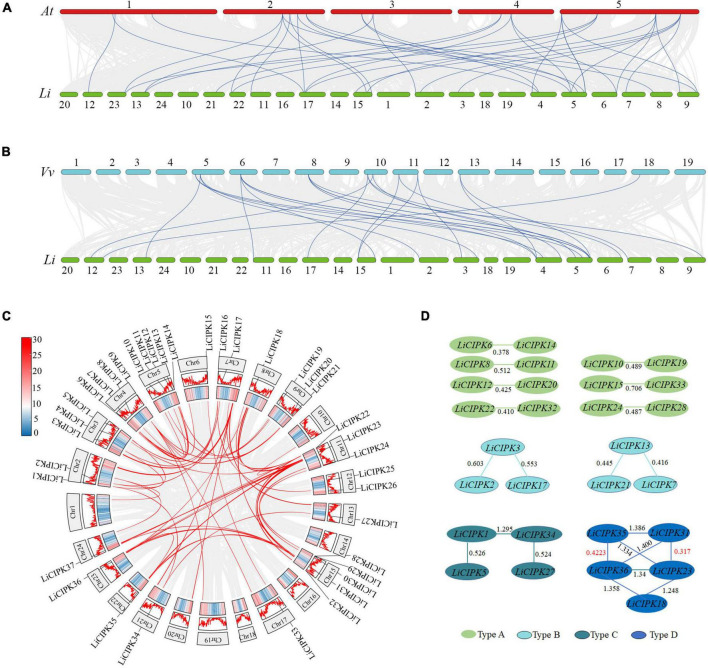
Inter- and intraspecies collinearity of *CIPK* loci. **(A)** *AtCIPKs* vs. *LiCIPKs*. **(B)** *VvCIPKs* vs. *LiCIPKs.* The light gray lines indicate the synteny between species, blue lines indicate cognate loci of *CIPKs.* **(C)** *LiCIPKs* vs. *LiCIPKs*. Red lines show synteny loci of *LiCIPKs*. Gene density across chromosomes are indicated by hot map (inner circle) and column map (medium circles), out circle show length of chromosomes. **(D)** Classification of duplicated *LiCIPK*. Light green, cyan, dark green, and blue indicate duplicated *LiCIPKs* containing two, three, four, and five members, respectively.

Due to an apparent increase in *LiCIPK* gene numbers, we further analyzed the inter-species collinear relationship of *CIPK* loci (*L. indica* vs. Arabidopsis, *L. indica* vs. grape). Our results showed that *CIPKs* loci were maintained differently during the evolution of the three species (Figure 4). As Figure 4 illustrates, only 10 *VvCIPK* and 15 *AtCIPK* collinear loci were identified in *L. indica* (Figures 4A,B). Meanwhile, various *CIPKs* were lost during evolution, including grape *VvCIPK33/30* and Arabidopsis *AtCIPK9* (*At1g01140*), which lack an orthologous gene in *L. indica* (Figure 4A). In contrast, *AtCIPK* and *VvCIPK* usually have more than one orthologous loci in *L. indica*. These results indicate that although some of the ancient CIPK have been lost, the remaining *LiCIPKs* have been duplicated during evolution (Figures 4A,B). As a result, the total number of *LiCIPK* gene family members is higher than that of grape and Arabidopsis.

To further elucidate the evolution of *LiCIPKs*, we surveyed cognate loci intraspecies, and discovered that 29 (78.38%) of the 37 *LiCIPKs* formed 21 duplicated pairs, which could be divided into four types according to their relationships (Figures 4C,D and Supplementary Figure 3). The type A duplicated genes contained two members, which was also observed in other plants such as grapes (Yin et al., 2017). Type B contained three members, however, these were not mutually duplicated members. Type C and D involved four (or more) members with complicated relationships. Types B–D have not been previously identified in other plants (Figure 4D). Additionally, the three loci harbored *LiCIPKs* very closely on chromosomes (*LiCIPK12* and *LiCIPK13 o*n chromosome 5; *LiCIPK20* and *LiCIPK21 o*n chromosome 9; *LiCIPK29* and *LiCIPK30 o*n chromosome 15), that were not tandem repeat loci (Supplementary Figure 3). This result coincides with the phylogenetic analysis, such as that LiCIPK30 has high similarity to *LiCIPK16*, but not to the adjacent *LiCIPK29* (Figure 3 and Supplementary Table 2).

The high percentage of duplicated *LiCIPKs* in *L. indica* prompted us to investigate the time of the duplication events. To this end, we calculated the *Ks* of *LiCIPK* duplicated pairs, and orthologous pairs between *LiCIPKs* and *AtCIPK*, *LiCIPKs* and *VvCIPKs* gene pairs. The results showed that *LiCIPK* paralogs have two apparent Ks peaks (Figure 4D and Supplementary Figure 4), indicating that the existing *LiCIPKs* experienced two duplication events (Table 2). According to the Myrtales specific molecular clock (1.14 × 10^–8^) reported previously (Feng et al., 2021), the two duplication events of *L. indica* were estimated to have occurred around 16.24–26.34 MYA and 56.12–61.16 MYA, respectively (Table 2). Interestingly, we found that type A and B duplicated genes were maintained by the recent duplication events, whereas type C and D duplicated genes experienced two duplicated events.

**TABLE 2:** Divergence time of CIPKs among three species.

Species – species		*Li – Li*	*Vv – Li*	*At – Li*
*K*_*a*_		0.0887 ± 0.0492	0.1857 ± 0.0722	0.2585 ± 0.0722
*K*_*s*_	Mean 1	0.485 ± 0.115	2.013 ± 0.735	2.664 ± 1.151
	Mean 2	1.337 ± 0.0574	–	–
*K*_*a*_/*K*_*s*_ (mean)		0.142 ± 0.072	0.103 ± 0.045	0.106 ± 0.071
Divergence time (MYA)	λ = 1.5 × 10^–8^ (mean 1)	16.18 ± 3.84	67.11 ± 24.62	88.99 ± 38.38
	λ = 1.14 × 10^–8^ (mean 1)	21.29 ± 5.05	88.23 ± 32.24	116.84 ± 50.48
	λ = 6.1 × 10^–9^ (mean 1)	39.79 ± 9.45	165.03 ± 62.31	218.33 ± 94.38
	λ = 1.5 × 10^–8^ (mean 2)	44.57 ± 1.91	–	–
	λ = 1.14 × 10^–8^ (mean 2)	58.64 ± 2.52	–	–
	λ = 6.1 × 10^–9^ (mean 2)	109.60 ± 4.70	–	–

All data are mean ± SD. *K*_*a*_, non-synonymous substitutions per synonymous; *K*_*s*_, synonymous substitutions per synonymous. MYA, million years ago.

The *K*_*s*_ value between *LiCIPKs–VvCIPKs* was 1.5–2, while that between *LiCIPKs–AtCIPK* was more than 2, implying that the *LiCIPKs* are more highly divergent from *AtCIPKs* than *VvCIPKs* (Supplementary Figure 4). This result is also consistent with the phylogenetic analysis (Figure 3). The average *K*_*a*_/*K*_*s*_ ratios of *LiCIPKs–VvCIPKs, LiCIPKs–VvCIPKs*, and *LiCIPKs* pairs are 0.103, 0.106, and 0.142, respectively. Hence, the *CIPKs* genes among the three species were under strong purifying selection (Table 2).

### *Cis*-elements in the promoter of *LiCIPKs*

**FIGURE 5:**
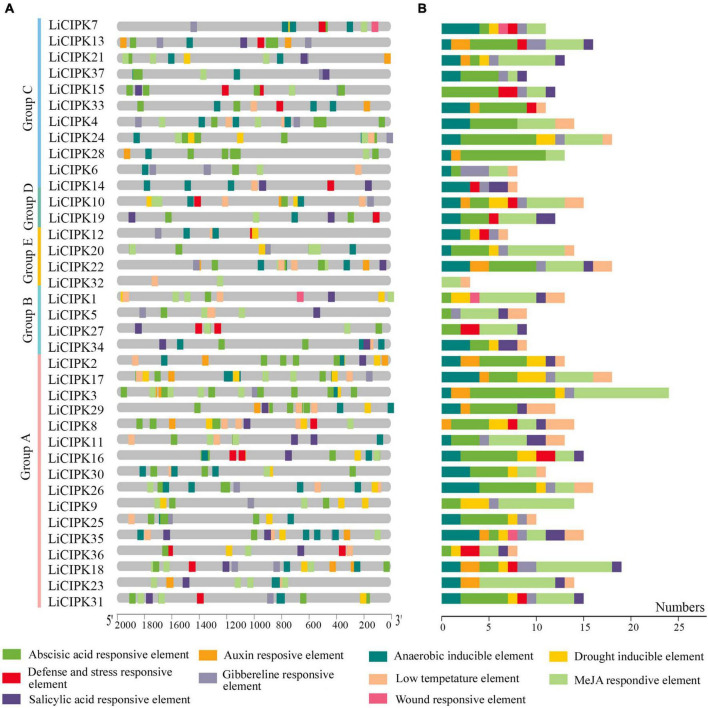
Distribution and numbers of abiotic stress and hormone responsive *cis*-elements in promoters of *LiCIPK*s. **(A)** The distribution of different *cis*-elements. **(B)** Numbers of different *cis*-elements. Elements are indicated as rectangles, others sequences as lines.

To clarify the regulatory mechanism of *LiCIPK* genes under abiotic stress, the *cis*-elements of the LiCIPKs promoters (−2,000 bp upstream ATG), which respond to plant hormones and abiotic stress were analyzed using PlantCARE software. The plant hormone and abiotic stress responsive elements were broadly distributed in *LiCIPK* promoters. Of these, the top three elements are ABRE (abscisic acid responsive element), MeJA (MeJA responsive element), and anaerobic inducible element (Figure 5). The overlapping of different elements on promoters is a common phenomenon. For example, defense, ABRE, GA, and auxin responsive elements were arranged in an array on the promoter of *LiCIPK13*, -*15*, -*34*, etc. From the *cis*-elements in the *LiCIPK* promoters, we deduced that *LiCIPKs* may be widely involved in plant hormone signaling and stress response. Furthermore, the number and location of *cis*-elements differed in the promoter of CIPK duplicated pairs, for example, *LiCIPK24* and 28 pairs (Figures 4, 5). These results imply functional differentiation of the duplicated genes (Figure 5).

### Different *LiCIPKs* respond to abiotic stress differently

**FIGURE 6:**
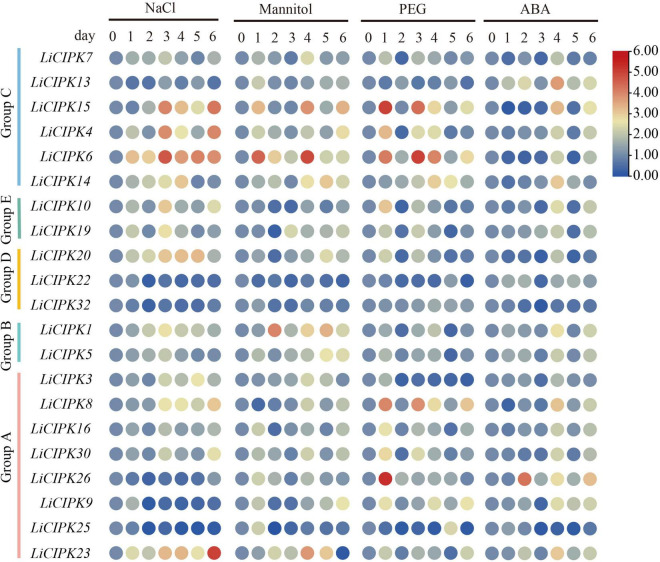
Expression profiles of *LiCIPKs* under four kinds of abiotic stress. Cuttings were treated by NaCl solution (200 mmol⋅L^–1^), mannitol solutions (200 mmol⋅L^–1^), 15% PEG8000 and ABA (10 × 10^–4^ mol⋅L^–1^), respectively. Roots were collected after treatment of 0, 1, 2, 3, 4, 5, and 6 days. The mean fold changes of the expression level are depicted by heat map. *n* ≥ 3.

To investigate the regulatory mechanism and potential function of LiCIPKs, we analyzed their expression profiles under NaCl, PEG ABA, and mannitol induced salt, drought, and osmotic stress using qRT-PCR. Of the 30 gene-specific primer pairs (Supplementary Table 1), 21 *LiCIPKs* were successfully amplified (Figure 6). This result indicates that under different abiotic stresses, expression levels of the responding members differ. Under salt stress, *LiCIPK4*, *−6*, and *−15* of group C; *LiCIPK10* and *−19* of group E; and *LiCIPK1* of group B reached their highest expression after 3 days of treatment, whereas *LiCIPK23* and −*30* peaked after 6 days of treatment. Under mannitol (osmotic) stress, *LiCIPK1* of group B and *LiCIPK4*, *−6*, and *−15* of group C exhibited fluctuating patterns during the 6 days of treatment. *LiCIPK15*, *−4, −6*, *−8*, and *−26* responded to PEG treatment more rapidly than to the other types of stress treatment. Most LiCIPKs responded to ABA until 4 days of treatment. Moreover, the expression patterns of certain members showed opposite tendencies, such as LiCIPK3 under salt and PEG treatment (Pearson *r* = −0.3659), and *LiCIPK1* under mannitol and PEG treatment (Pearson *r* = −0.6001). The members *LiCIPK4*, *−6*, *−15*, *−14*, −*10*, *−1*, *−5*, *−8, −16*, *−30*, −9, and −23 responded to all four stress treatments, indicating that they may be involved in stress signaling interplay. However, other members, including *LiCIPK22*, *−2*5, and −32 exhibited relatively no changes under the four treatments (fold changes < 2) and thus, members did not likely participate in the stress response under our experimental conditions.

### *LiCIPK30* complements *AtSOS2* in Arabidopsis

**FIGURE 7:**
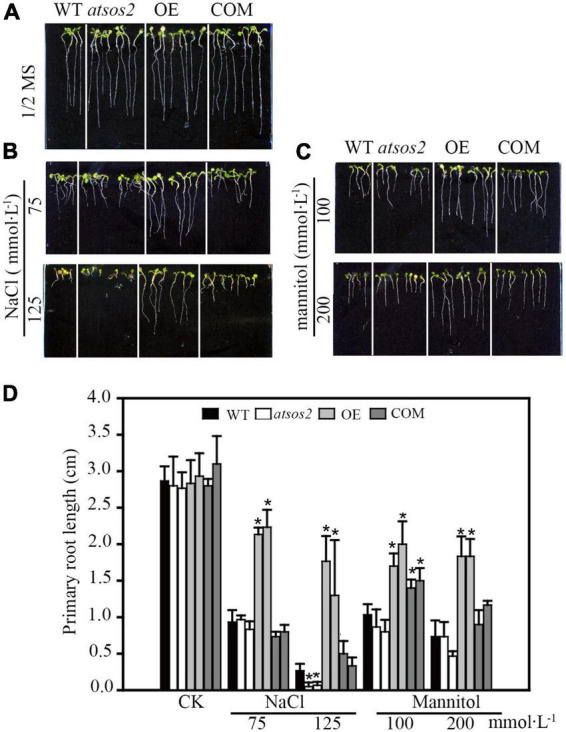
Phenotype of WT, *atsos2*, OE, and COM under salt and mannitol stress. **(A)** 1/2 MS. **(B)** 1/2 MS with 75, 125 mmol L^–1^ NaCl. **(C)** 1/2 MS with 100 and 200 mmol L^–1^ mannitol. **(D)** Primary root length. Data were collected from seeds grown on medium 10 days after planting. Mean ± SD, *n* ≥ 3, *p < 0.05 (Student’s *t*-test).

Phylogenetic analysis showed that LiCIPK30 is an orthologous gene of AtCIPK24 (AtSOS2) (Figure 3) and responds to the four abiotic stresses (Figure 6). However, whether LiCIPK30 is a bona fide SOS2 gene requires verification. To clarify the function of LiCIPK30 *in vivo*, we developed a 35S:LiCIPK30 construct, and transformed it into the *atsos2* mutant and Arabidopsis WT (Col-0). After genotype identification and expression analysis of the transformed lines, the complementary lines (COM) and over-expression lines (OE) were successfully obtained (Supplementary Figures 5, 6). The seeds of the four lines (WT, *atsos2*, OE, and COM) were germinated under salt (0, 75, and 125 mmol L^–1^ NaCl) and osmotic (0, 100, and 200 mmol L^–1^ mannitol) stress conditions. After 10 days, the primary root length of all four lines showed no difference under normal conditions (1/2 MS; Figure 7A). Under salt stress, OE lines exhibited the highest growth rate, while atsos2 had the lowest, and that of COM and WT lines were between OE and the mutant, however, were all inhibited (Figure 7). Under osmotic stress, WT, *atsos2*, OE, and COM lines have similar phenotype as that of salt stress (Figure 7). Collectively, LiCIPK30 could salvage the salt- and osmotic-sensitive phenotype of *atsos2*. In fact, over-expression of LiCIPK30 in WT enhanced salt/osmotic tolerance of Arabidopsis during the germination and seedling stages. By combining the results of phylogenic analysis, we refer to LiCIPK30 as LiSOS2 hereafter.

### The physiological mechanism of LiSOS2 confers stress tolerance of Arabidopsis

**FIGURE 8:**
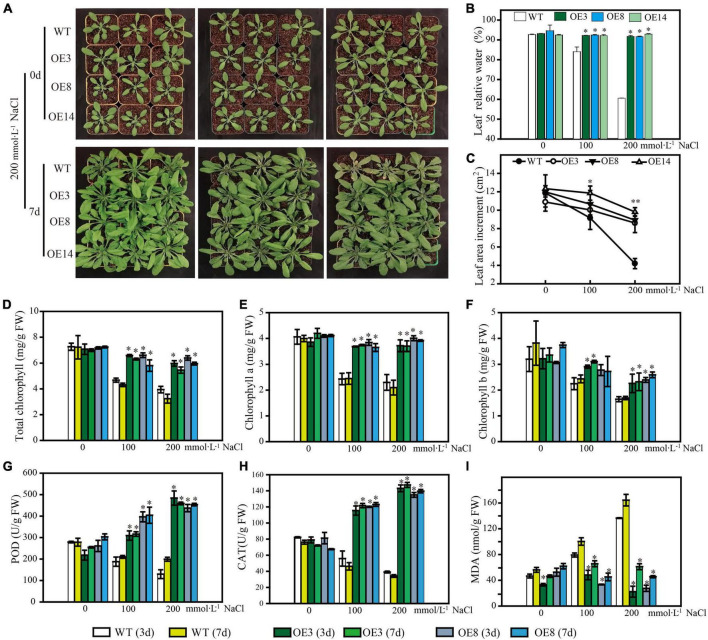
Phenotype of LiSOS2 OE lines. **(A)** Phenotype of plants before and after salt stress. **(B)** Relative water contents of leaves. **(C)** Leaves area increment per plant after salt treatment. **(D)** Total content of chlorophyll. **(E)** Chlorophyll a. **(F)** Chlorophyll b. **(G)** Activity of POD. **(H)** Activity of CAT. **(I)** Content of MAD. Mean ± SD, *n* ≥ 3, *p < 0.05, ^**^p < 0.01 (Student’s *t-*test, compared to the WT at same conditions).

To further uncover the function of LiSOS2, we observed the phenotype of OE lines during the rosette leaf stage under different salt stress conditions (0, 100, and 200 mmol L^–1^ NaCl). All OE lines maintained growth well under stress conditions, while WT growth appeared to be inhibited with lower relative water content and smaller leaf area after 7 days of salt treatment (Figures 8A–C). The total chlorophyll content (chlorophyll a and b) appeared to decrease in WT and decreased weakly in the OE lines under 200 mmol L^–1^ NaCl treatment (no significant statistical difference) (Figures 8D–F). Moreover, the activity of reactive oxygen species (ROS) scavenging enzymes, POD, and CAT increased in the OE lines and decreased in WT (Figures 8G,H). MDA content increased under the harsher salt treatment (increased concentration and prolonged time) in WT and decreased in OE lines (Figure 8I). Collectively, these results indicate that the overexpression of LiSOS2 in Arabidopsis confers salt stress tolerance through developmental adaptation (regulating leaf size), decreased damage to the leaf photosynthetic system, membrane lipid peroxidation, and enhanced ROS scavenging ability (physiological adaptation).

### LiSOS2 enhances the expression of *AtABF3* and *AtRD22 in vivo* under salt stress

**FIGURE 9:**
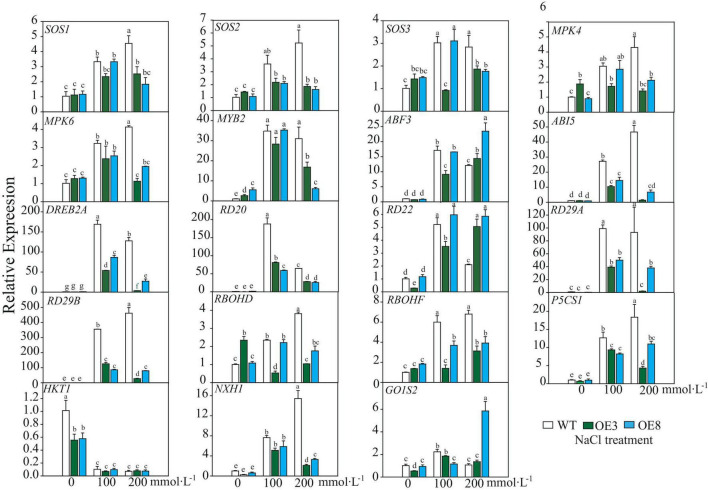
Relative expression of salt stress responsive genes of WT and OE lines. *SOS*, Salt Overly Sensitive; MPK, mitogen-activated protein kinas; MYB, V-MYB avian myeloblastosis viral oncogene homolog; ABF, ABA response element binding factor; ABI, abscisic acid insensitive, DREB, dehydration responsive element binding proteins 2; RD, responsive to desiccation; RBOH, respiratory burst oxidase homolog; P5CS, ^Δ^1-pyrroline-5-carboxylate synthetase; HKT, high-affinity potassium transporter; NHX, Na^+^/H^+^ antiporter; GolS, galactinol synthase. Mean ± SD, *n* = 3, lowercase letters indicate significant difference between samples (*p* ≤ 0.05, two ways ANOVA).

After the plant perceives a salt stress signal, it responds through an interplay of several pathways to decrease the detrimental effect. Hence, we detected expression profiles of the endogenous genes, including those of the SOS pathway (*SOS1–SOS3*), mitogen-activated protein kinase (which functions through ABA pathway), ABA-dependent signaling pathway (*ABF3*, *ABI5*, *RD22*, *RD29A*, and *RD29B*), ABA-independent signaling pathway (*DREB2A*, *RD20*, and *RD29A*), ROS signal (respiratory burst oxidase homolog, RBOH), and ion homeostasis (Na^+^. K^+^), as well as small molecular proline biosynthesis-related gene and membrane signal-related gene *GOlS2* (galactinol synthase 2). These genes, excluding *AtHKT1*, were induced under salt stress in both WT and OE lines. It was also evident that the fold changes of these induced genes differed between WT and OE lines. Among the 18 upregulated genes, only *ABF3, AtRD22*, and *GOlS2* expressions in OE were higher than that of WT under higher salt stress (Figure 9). According to the induced expression patterns in WT, the upregulated genes could be divided into three classes: Class I, genes that were continuously induced as salt stress was enhanced, namely, *SOS2*, *MPK4*, *MPK6*, *ABI5*, *RD29B*, *RBOHD*, *P5CS1*, and *NHX1*. Class II, genes induced under lower salt stress (100 mmol L^–1^ NaCl) but downregulated under 200 mmol L^–1^ NaCl stress, namely, *ABF3*, *DREB2A*, *RD20*, and *RD22*. Class III, invariable genes, namely, *SOS3*, *MYB2*, *RD29A*, *RBOHF*, and *GOlS2* (Figure 9). Additionally, the expression of most genes in the OE lines remained relatively invariable under the two stress conditions, indicating that these genes may be regulated under invariable signals. Based on these results, we concluded that under the “protection” of LiSOS2, OE plants did not respond as strongly to harsh stress conditions as WT.

## Discussion

In this study, we surveyed the *CIPK* gene family of the ornamental plant *L. indica* through mining recently sequenced reference genome data (manuscript under preparation). Our results indicated that there are 37 *LiCIPKs* in *L. indica* that can be divided into two classes according to their intron/exon patterns, or five groups according to the phylogenic relationship of Arabidopsis, grape, rice, and *L. indica CIPKs* (Figures 1, 3). The intron-rich (43.25%) and intron-less (56.75%) patterns of *LiCIPK* genes were similar to that of *AtCIPK*s, *VvCIPKs*, and *OsCIPKs* (Kolukisaoglu et al., 2004; Kanwar et al., 2014; Xi et al., 2017). Besides these four species, the exon/intron structure of *CIPKs* of *Populus* (Yu et al., 2007), maize (Chen et al., 2011), canola (Zhang et al., 2014), and pepper (*Capsicum annuum* L.) (Ma et al., 2019) were highly similar. These results suggest that the diversity of *CIPK* gene structure predated the split of angiosperms. A previous investigation indicated that intron-less CIPKs first appeared in the basal angiosperm *Amborella trichopoda* and were derived from retrotransposition events that occurred in the ancestor of angiosperm plants (Kleist et al., 2014; Zhang et al., 2020). Hence, the gene structure of *LiCIPKs* was inherited from their ancestor species.

To date the reported CIPK gene family members in plant genomes have been under pure selection (Zhang et al., 2014; Sun et al., 2015; Li et al., 2016; Xi et al., 2017; Yin et al., 2017; Liu et al., 2019), indicating relatively conserved amino acid sequences among plant *CIPKs*. In this study, the 37 *LiCIPKs* could be divided into several groups according to their phylogenetic relationships, conserved motifs, and 3D structures. Regarding kinase function, we considered that information from 3D-structures may be more credible than that from MEME motifs. From the regulatory mode of AtCIPK (Chaves-Sanjuan et al., 2014), we deduced that class A–E LiCIPKs depend on CBL to switch on and PP2C (or other types of phosphatase) to switch off kinase activity, due to their compact 3D-structure (Figure 2 and Supplementary Figure 2). The class F LiCIPKs may have higher basal kinase activity due to their open structure, however, the full activity requires binding to a specific CBL (Figure 2 and Supplementary Figure 2). Few LiCIPKs lacked the C-lobe, which was not anticipated as their NAF/FISL and PPI were intact (Figures 1, 2 and Supplementary Figures 1, 2). The reason for this may be the divergent sequences at the C-terminal. However, the N-terminal 3D-structures of LiCIPKs highly resemble those of AtCIPK24 (Chaves-Sanjuan et al., 2014). We speculate that the kinase activity of these types of LiCIPKs still depend on CBL, however, is independent of phosphatase. In addition, the serine insertion in the NAF/FISL motif of LiCIPK6 and -14 increases the hydrophilicity of these regions (Supplementary Figure 1), which may decrease binding activity between CBL and CIPKs according to the interactions of AtCIPK24/SOS2 and AtCBL4/SOS3 (Sánchez-Barrena et al., 2007). Hence, future work should identify the CBL partner, kinase activity and downstream targets.

The most striking evolutionary characteristic of *LiCIPKs* is the high percentage (∼80%) of duplicated *LiCIPKs* in the *L. indic*a genome (Figures 4C,D and Supplementary Figure 3). This indicates that they are the vestiges of WGD events, not of chromosome segmental duplication events. From the two separate *K*_*s*_ peak distributions (Supplementary Figure 4), combined with the results of previous investigations (Myburg et al., 2014; Qin et al., 2017; Luo et al., 2020; Feng et al., 2021), we inferred that the older WGD1 event (56.12–61.16 MYA) occurred commonly in the *Lythraceae* ancestor, while the recent WGD2 event (16.24–26.34 MYA) may have occurred exclusively in *Lagerstroemia* species (Table 2). Our results are supported by the phylogenomic analysis of 20 *Lagerstroemia* chloroplast genomes, which shows that *Lagerstroemia* originated in the late Paleocene (∼60 MYA), and *Lagerstroemia* species were thriving around 11.8–31.6 MYA (Dong et al., 2021). However, currently, only one genome dataset exists for *Lagerstroemia*, whether this WGD event occurred commonly in *Lagerstroemia* species still requires verification.

It is well known that dicotyledon experienced three common WGD events: ζ, ε, and γ (Tang et al., 2008; Clark and Donoghue, 2018; One Thousand Plant Transcriptomes, 2019; Li and Barker, 2020). As for *L. indica*, it experienced five WGD events contained two WGD events we uncovered in this research, hence, it is interesting how the *LiCIPKs* were retained after five WGD events. Based on the phylogenic relationships of CIPKs among Arabidopsis, gape, rice and *L. indica*, duplicated pairs of *LiCIPKs* (Figures 3, 4, Table 2, and Supplementary Table 2), and the previous report (Zhang et al., 2020), we deduced the evolutionary history of *LiCIPKs* (Supplementary Figure 7). Retaining of LiCIPKs after two linkages specific WGDs were summarized in Supplementary Table 3. After two WGD events and genome reshuffling, different genes numbers were kept. We deduced that the types A–C may have originated from one ancestral gene, but type D cluster has two segmental duplicated ancestral genes. In type A, only two WGD2 duplicated members were retained; in type B, one of the WGD1 duplicated members was lost; In type C, all four genes were retained; in type D, three WGD1 members and two WGD2 members were retained, however, collinearity of five loci was more intact than that of loci of types A–C, genes involved have multilateral relationships. Apart from the duplication pairs, the eight *LiCIPKs* (∼20%) lacking duplicated pairs were likely the members retained in the genome by ancient WGDs, or their duplicated paralogous were lost (Supplementary Figure 7). Interestingly, their orthologous pairs in Arabidopsis experienced similar evolutionary mechanisms, for example, *AtCIPK6, AtCIPK8*, *AtCIPK23*, and *AtCIPK24*/*SOS2* were not expanded (or their paralogs were lost) after linkage specific α, and β WGD events and they are the retained members following ancient WGD event (Zhang et al., 2020). Gene balance hypothesis have been widely accepted to explain genes retaining after WGD (Birchler and Veitia, 2021), future work should be undertook to identify the specific interaction LiCBL-LiCIPK and their function in *L. indica*.

In this study, we surveyed the *cis*-elements of *LiCIPK* promoters and detected their expression through qRT-PCR (Figures 5, 6). We found that expression levels and the numbers of several *cis*-elements were not positively correlated, which may have been due to gene expression depending on the co-operation between *cis*-elements and *trans*-factors (such as TFs, RNA Pol, etc.) and the post-transcription regulation mechanism. As kinases, CIPKs regulate activities of several downstream proteins, for instance, MdCIPK22 regulates MdAREB2 in apples (Ma et al., 2017). However, since the transcriptional monitor of CIPKs received little attention, future studies will aim to identify the factors affecting *LiCIPK* expression under abiotic stress, which will facilitate a comprehensive elucidation of the complete regulatory mechanism of CIPKs. Additionally, the variety we used in this study differs from used for genome sequencing, hence, sequence polymorphisms of the promoter may also account for the difference between the predicted and observed results.

Comparing the expression of *LiCIPK30*/*SOS2* in *L. indica* (Figure 6) and *AtCIPK24*/*SOS2* in Arabidopsis (Figure 9), we found that *LiCIPK30* is not induced like *AtCIPK24*/*SOS2* under salt stress (Figures 6, 9). This may be a key difference between the two species. The *L. indica* variety (Black Diamond ‘Blush V2’) we used in this study is salt tolerant, with the ability to grow new buds and roots under 75 mmol⋅L^–1^ NaCl stress (unpublished lab data). Hence, under the conditions of the current study, we deduced that it may not have sensed the high stress, causing the dissimilarity in the expression patterns of *LiCIPK30*/*SOS2* to that of Arabidopsis (Figure 9). Based on the phenotype of OE Arabidopsis, we inferred that upregulation of *LiCIPK30*/*SOS2* expression protects plants from the detrimental impact of salt stress through developmental adaptation and physiological adaptation (Figures 7, 8). The function of *LiCIPK30* in Arabidopsis may also partially occur through the ABA pathway as both *AtRD22* and *AtABF3* are ABA-responsive genes (Figure 9) (Yoshida et al., 2010; Liu et al., 2018). *ABF3* overexpression confers tolerance to multiple abiotic stresses in alfalfa (Wang et al., 2016) and drought tolerance in Arabidopsis and rice (Oh et al., 2005; Yoshida et al., 2010). However, currently the mechanism by which *LiCIPK30*/*SOS2* regulates the transcription of *ABF3* is unclear. Nevertheless, yeast-two-hybrid and BiFC assays have revealed that VaCIPK02 of amur grape modulates ABA signaling through interacting with the ABA receptor-PYL9 (Xu et al., 2020). Hence, it is possible that *LiCIPK30*/*SOS2* activates the components upstream of *ABF3*. Elevated expression of *GOlS2* may lead to an increase in raffinose accumulation, which serves as an osmotic compound *in vivo*. The GOlSs have been reported to confer abiotic stress, particular to drought and cold (Li et al., 2020c; Liu Y. et al., 2020; Dai et al., 2022). Collectively, the enhanced Na^+^ exclusion, ABA pathway signaling, and ROS scavenging, as well as small osmotic compounds, coordinate to improve the performance of OE plants under salt and osmotic stress.

## Conclusion

Our data reveal the characteristics and evolutionary history of *LiCIPKs*, as well as the gene resources involved in abiotic stress. Ectopic expression of *LiCIPK30* in Arabidopsis enhances salt stress tolerance. This work also advances the current understanding regarding the complex interaction between *L. indica* and its harsh environmental conditions. Further studies are required for an in-depth elucidation of these interactions.

## Data availability statement

The datasets presented in this study can be found in online repositories. The names of the repository/repositories and accession number(s) can be found in the article/Supplementary material.

## Author contributions

CY and JZ contributed to conception and design of the study. YK, YH, YZ, YL, HW, GL, BL, YC, and FZ performed the experiments. CY and YK prepared the manuscript. All authors contributed to the article and approved the submitted version.

## Footnotes

1
https://pfam.xfam.org

2
http://web.expasy.org/protparam/

3
https://wolfpsort.hgc.jp/

4
http://www.cbs.dtu.dk/services/SignalP/

5
http://meme-suite.org/tools/meme

6
https://swissmodel.expasy.org

7
http://www.cgl.ucsf.edu/chimera/download.html

8
https://evolgenius.info/

9
http://bioinformatics.psb.ugent.be/webtools/plantcare/html/

## Supplementary material

Supplementary Figure 1 Multi-alignment of LiCIPKs N and C conserved motifs. The top is the logo of the conserved amino acids. The red boxes show the amino acid residues may affect the interaction between CBL sensor.

Supplementary Figure 2 The three-dimensional (3D) structure of 37 LiCIPKs. **(A–E)** Member with 3D Structure reassemble AtCIPK24/SOS2. The “a”, “b,” and “c” indicates three α-helixes ahead the NAF domain, respectively. **(F)** Members have a looser structure. **(G,H)** Members only with N-terminal 3D structure. Member of class G is monomer, of class H is homo-dimer.

Supplementary Figure 3 Chromosome location of *LiCIPKs*. Chromosome length show on left. Blue lines show segmental duplication of LiCIPKs.

Supplementary Figure 4 *K*_*s*_ of synteny CIPKs pairs of three species. *Li, L. indica; At*, *Arabidopsis thaliana*; *Vv*, Vitis vinifera. ND, genes pairs are too divergence to detect.

Supplementary Figure 5 Identification of *LiCIPK30* OE Arabidopsis. **(A)** Genotype of different single plant. Lane M: DL2,000. Lanes 1–3, WT, negative control. Lanes 4–22 different individual plant with hygromycin resistance. *LiCIPK30* specific primers were designed for PCR detection the genotype of the individual plant. PCR products were separated by 1% agarose gel. Positive OE plants were recoded (white number). **(B)** qRT-PCR detected the expression of *LiCIPK30.*

Supplementary Figure 6 Identification of atsos2 mutant *and LiCIPK30* COM lines. **(A)** Identification of *atsos2* mutant. Lanes labeled by odd numbers are PCR products of SALK_056101 specific forward and reverse primers (LP and RP), lanes labeled by even numbers are PCR products of LBb1.3 (BP) and RP. **(B)** Genotype of COM lines. **(C)** qRT-PCR detected the expression of *LiCIPK30.*

Supplementary Figure 7 Phylogenic tree of LiCIPKs. Whole genome duplication (WGD) events of angiosperm-plants are indicated on the branches (ζ, zeta seed plant-wide WGD; ε, epsilon angiosperm-wide WGD event; and γ, gamma triplicated of dicotyledon-wide WGD). The “T” indicates tandem repeat duplication happened in the ancestor of *L. indica.* WGD1 and WGD2 indicate two WGD events in *L. indica*.

## Abbreviations

CIPKs: CBL-interacting protein kinases
ROS: reactive oxygen species
CBLs: calcineurin B-like proteins
SOS: salt over sensitive
WGD: whole genome duplication
WT, Arabidopsis: Col-0
T-DNA SALK_056101C: *atsos2* mutant
COM: LiCIPK30/*atsos2*
OE: LiCIPK30/WT
qPCR: quantitative polymerase chain reaction
pI: isoelectric point
MW: molecular weight
NJ: neighbor-joining
CAT: catalase
POD: peroxidase
MAD: malondialdehyde
RBOH: respiratory burst oxidase homolog
*GOlS2*: galactinol synthase 2
PPI: protein phosphatase interaction.
