# Supplementary figures and images for: Genome-wide identification of calcineurin B-like protein-interacting protein kinase gene family reveals members participating in abiotic stress in the ornamental woody plant Lagerstroemia indica
Source: Front Plant Sci. 2022 Sep 20;13:942217. doi: 10.3389/fpls.2022.942217 (PMC9530917; doi:10.3389/fpls.2022.942217)

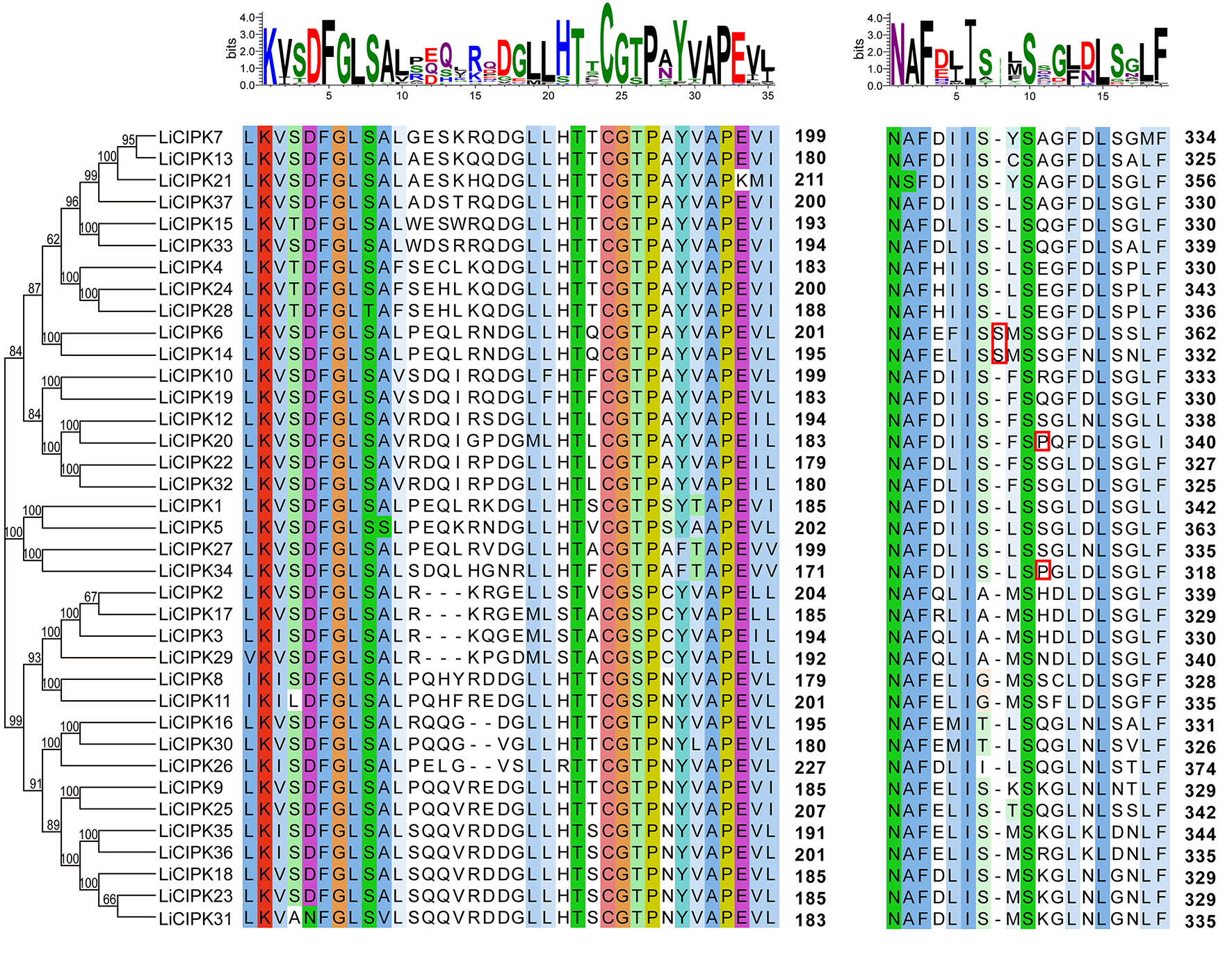

Supplement: Supplementary Figure 1 — Multi-alignment of LiCIPKs N and C conserved motifs. The top is the logo of the conserved amino acids. The red boxes show the amino acid residues may affect the interaction between CBL sensor. [file Image_1.TIF]

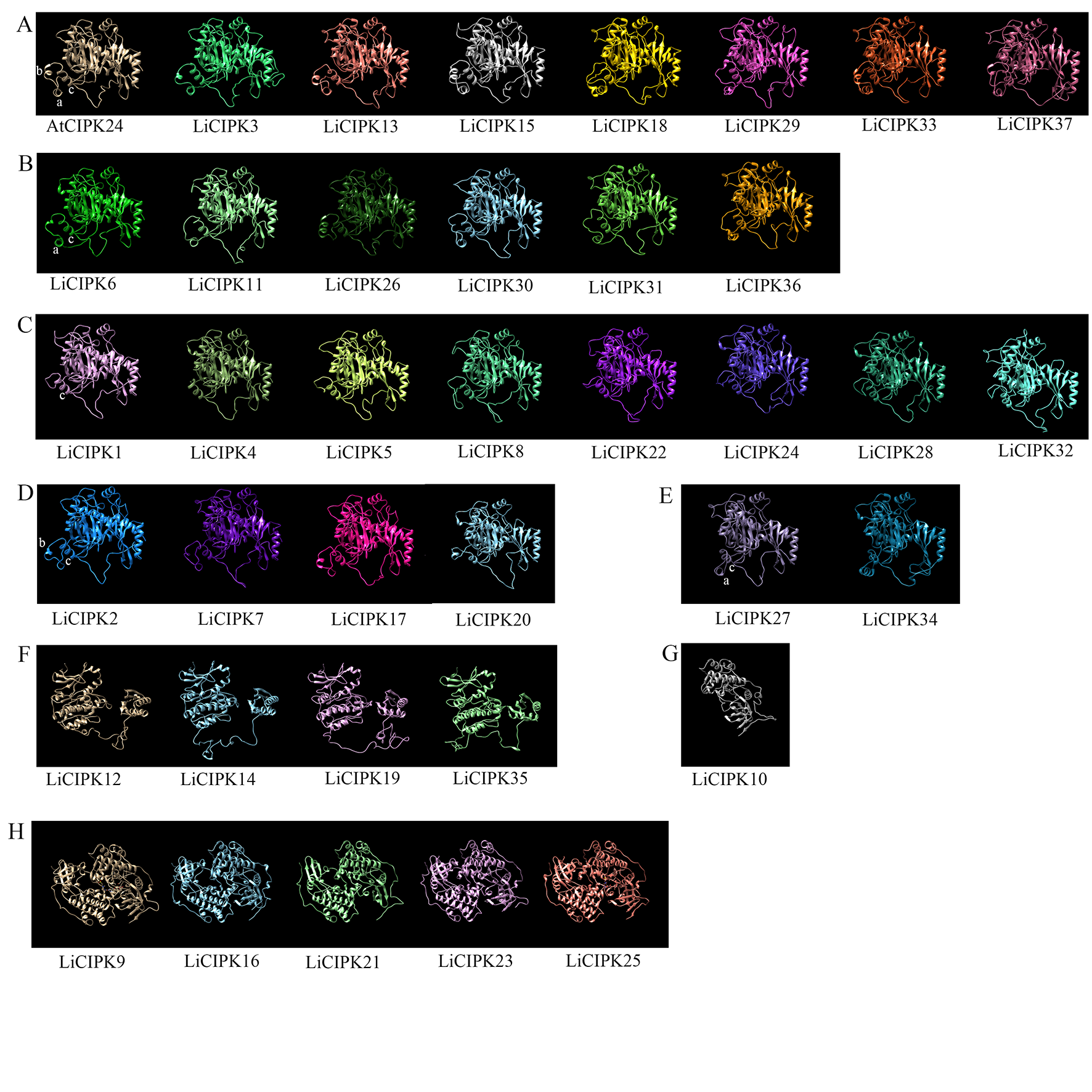

Supplement: Supplementary Figure 2 — The three-dimensional (3D) structure of 37 LiCIPKs. (A–E) Member with 3D Structure reassemble AtCIPK24/SOS2. The “a”, “b,” and “c” indicates three α-helixes ahead the NAF domain, respectively. (F) Members have a looser structure. (G,H) Members only with N-terminal 3D structure. Member of class G is monomer, of class H is homo-dimer. [file Image_2.TIF]

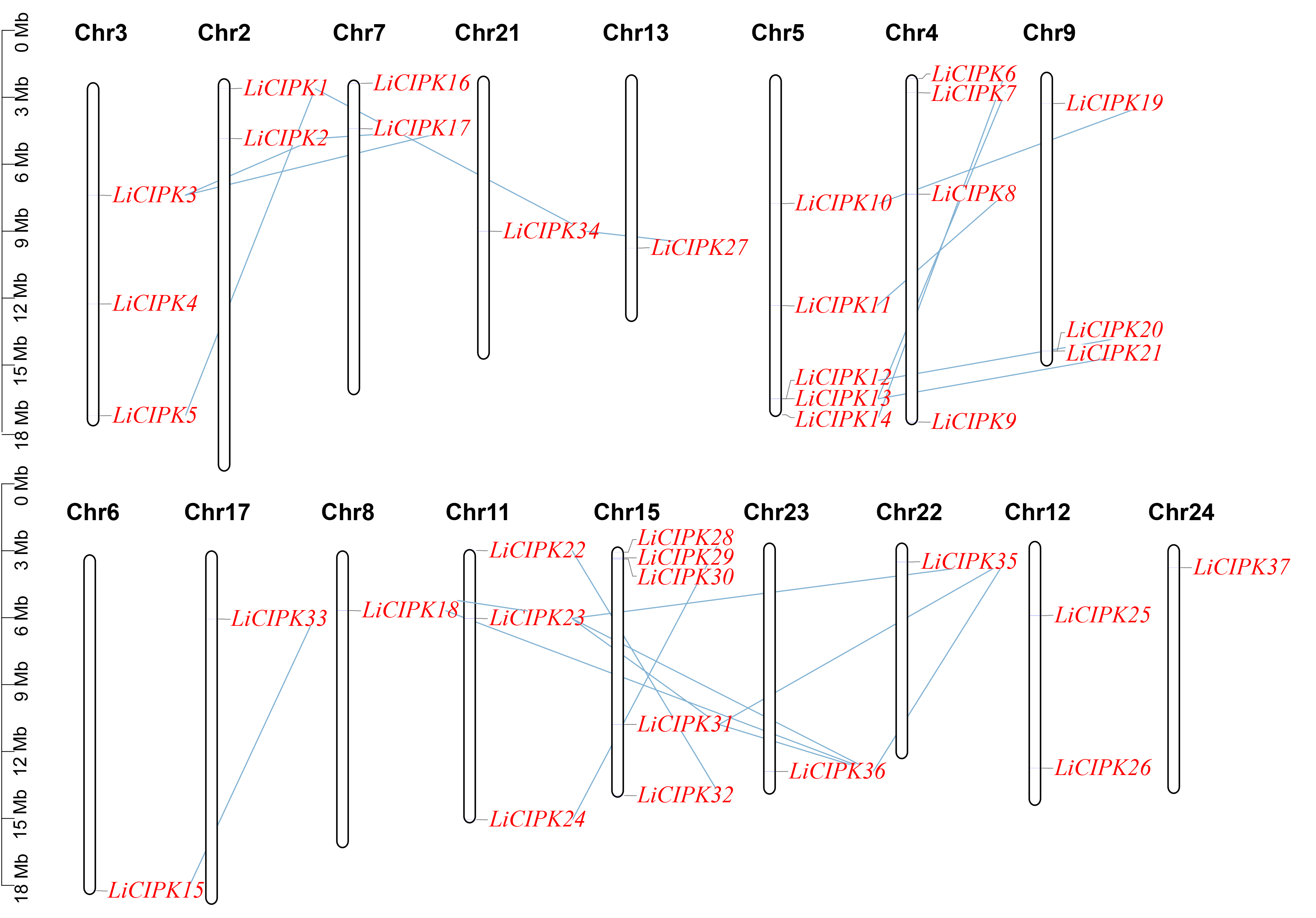

Supplement: Supplementary Figure 3 — Chromosome location of LiCIPKs. Chromosome length show on left. Blue lines show segmental duplication of LiCIPKs. [file Image_3.JPEG]

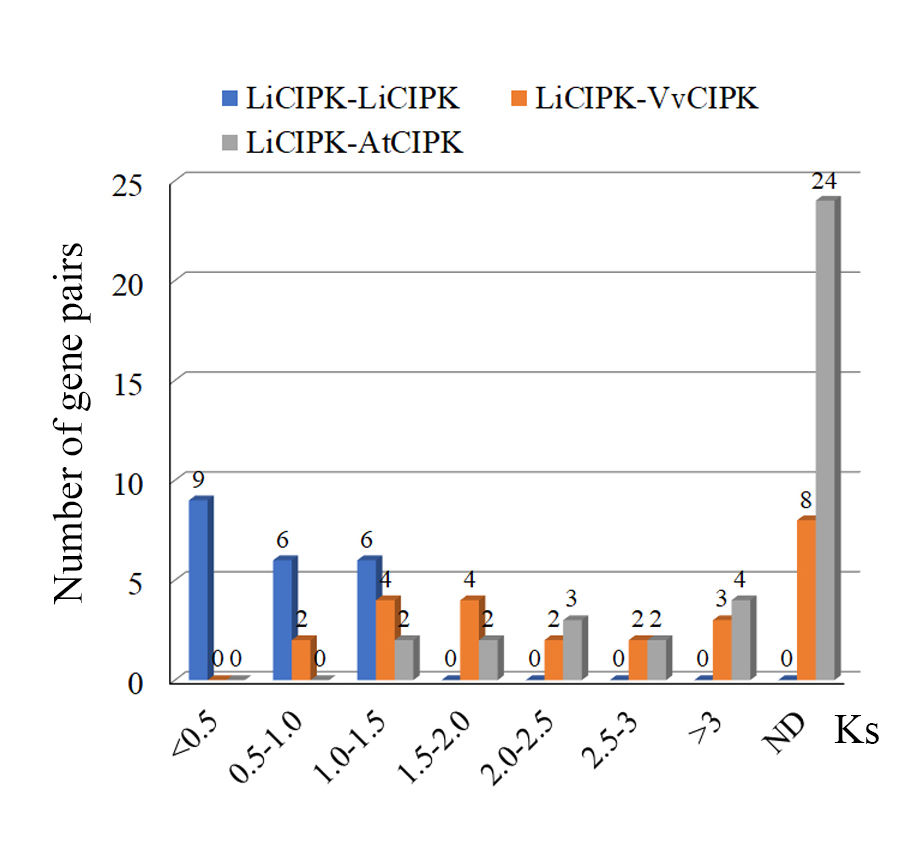

Supplement: Supplementary Figure 4 — Ks of synteny CIPKs pairs of three species. Li, L. indica; At, Arabidopsis thaliana; Vv, Vitis vinifera. ND, genes pairs are too divergence to detect. [file Image_4.JPEG]

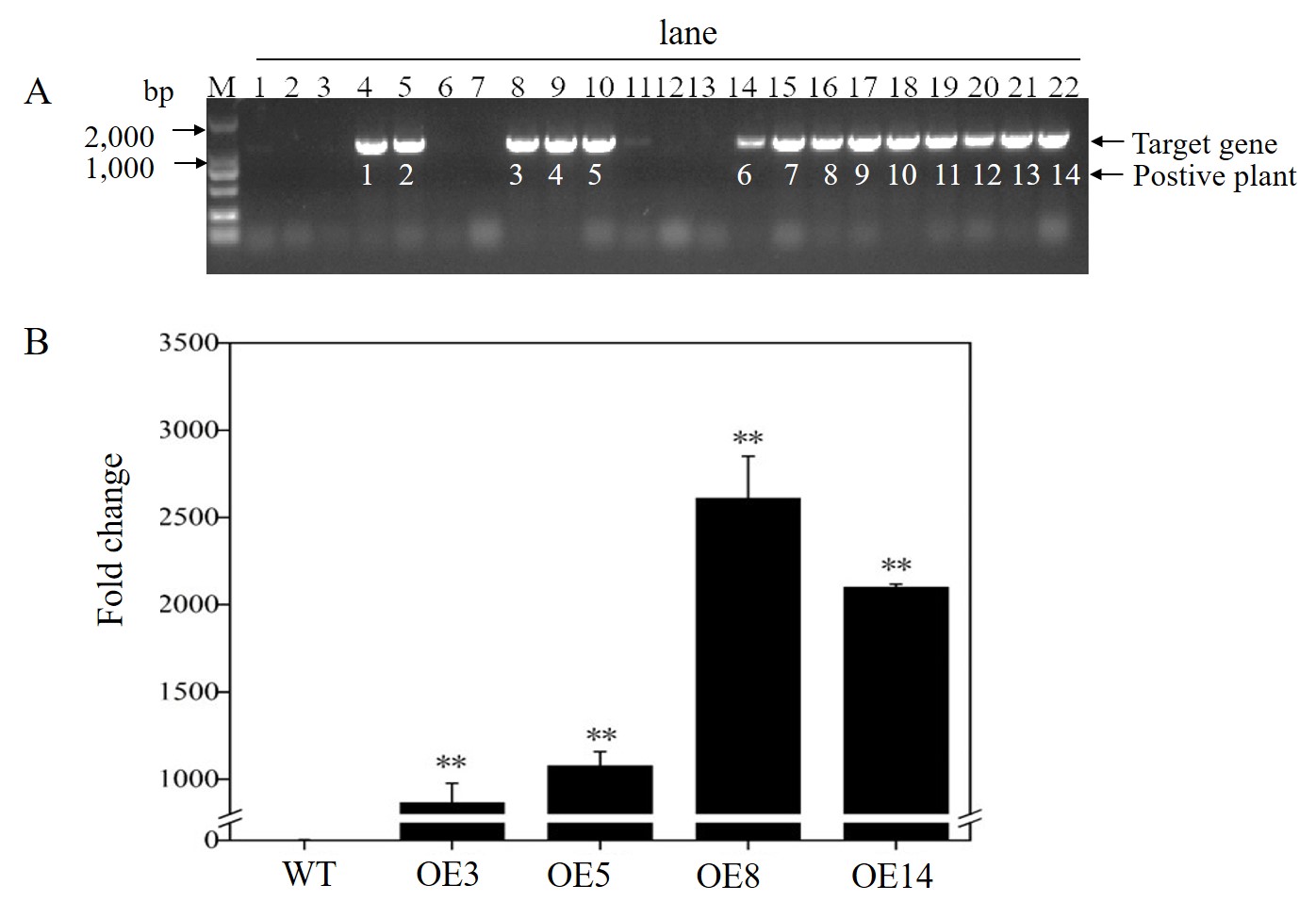

Supplement: Supplementary Figure 5 — Identification of LiCIPK30 OE Arabidopsis. (A) Genotype of different single plant. Lane M: DL2,000. Lanes 1–3, WT, negative control. Lanes 4–22 different individual plant with hygromycin resistance. LiCIPK30 specific primers were designed for PCR detection the genotype of the individual plant. PCR products were separated by 1% agarose gel. Positive OE plants were recoded (white number). (B) qRT-PCR detected the expression of LiCIPK30. [file Image_5.JPEG]

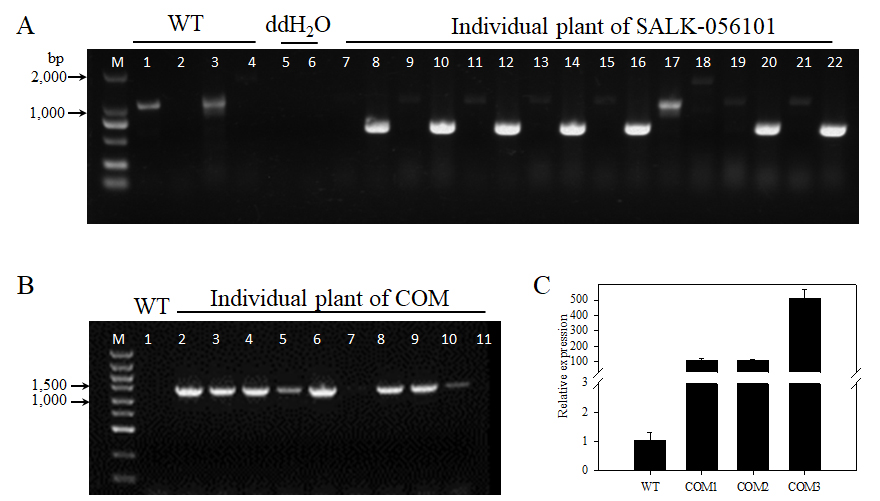

Supplement: Supplementary Figure 6 — Identification of atsos2 mutant and LiCIPK30 COM lines. (A) Identification of atsos2 mutant. Lanes labeled by odd numbers are PCR products of SALK_056101 specific forward and reverse primers (LP and RP), lanes labeled by even numbers are PCR products of LBb1.3 (BP) and RP. (B) Genotype of COM lines. (C) qRT-PCR detected the expression of LiCIPK30. [file Image_6.jpg]

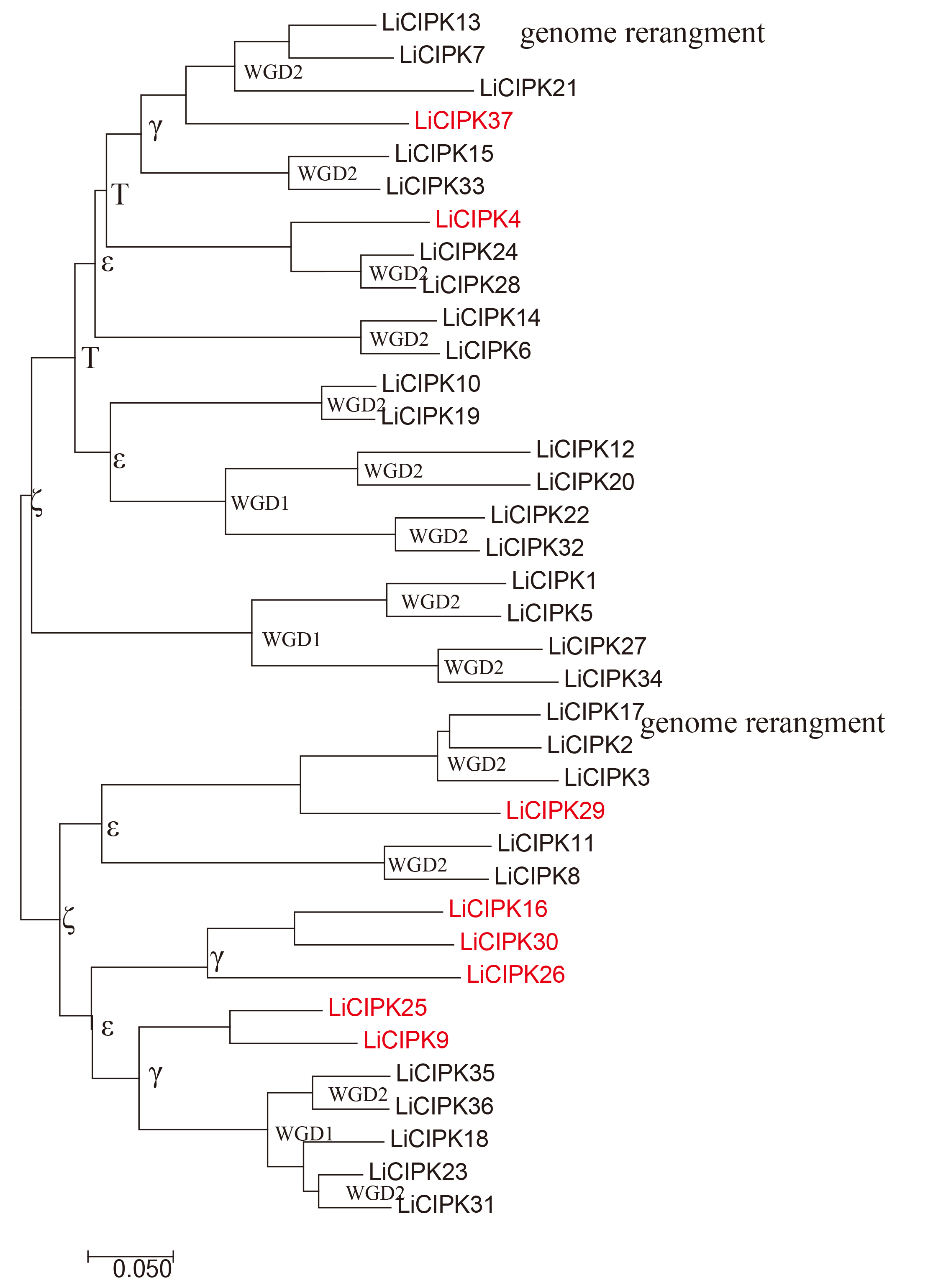

Supplement: Supplementary Figure 7 — Phylogenic tree of LiCIPKs. Whole genome duplication (WGD) events of angiosperm-plants are indicated on the branches (ζ, zeta seed plant-wide WGD; ε, epsilon angiosperm-wide WGD event; and γ, gamma triplicated of dicotyledon-wide WGD). The “T” indicates tandem repeat duplication happened in the ancestor of L. indica. WGD1 and WGD2 indicate two WGD events in L. indica. [file Image_7.jpg]
