# Supplementary material for: Genome-wide identification of calcineurin B-like protein-interacting protein kinase gene family reveals members participating in abiotic stress in the ornamental woody plant Lagerstroemia indica
Source: Front Plant Sci. 2022 Sep 20;13:942217. doi: 10.3389/fpls.2022.942217 (PMC9530917; doi:10.3389/fpls.2022.942217)
Supplement: Supplementary file 10 [file Table_3.DOCX]

**Supplementary Table 3** | Numbers of LiCIPKs retaining after two linkage specific WGDs of four different duplicated pairs

| Type of duplicated pairs* | Number of ancestral loci | WGD1 | WGD2 |
| --- | --- | --- | --- |
| Type A | 1 | 0 | 2 |
| Type B | 1 | 0 | 3 |
| Type C | 1 | 2 | 2 |
| Type D | 2 | 3 | 2 |

* Details of duplicated pairs are depicted in Figure 4D.
